# Knowledge, attitude, and its correlates of the community toward mental illness in Mattu, South West Ethiopia

**DOI:** 10.3389/fpsyt.2022.1018440

**Published:** 2022-11-08

**Authors:** Mohammedamin Hajure Jarso, Gebiso Roba Debele, Wubishet Gezimu, Desalegn Nigatu, Mustefa Mohammedhussein, Aman Mamo, Aman Dule, Mohammedjud Hassen, Kiyar Jemal

**Affiliations:** ^1^Department of Psychiatry, College of Health Sciences, Mattu University, Mattu, Ethiopia; ^2^Department of Public Health, College of Health Sciences, Mattu University, Mattu, Ethiopia; ^3^Department of Nursing, College of Health Sciences, Mattu University, Mattu, Ethiopia; ^4^Department of Psychiatry, College of Health Sciences, Madda Walabu University, Bale, Ethiopia; ^5^Department of Nursing, College of Health Sciences, Madda Walabu University, Shashemene, Ethiopia; ^6^Department of Health Informatics, College of Health Sciences, Mattu University, Mattu, Ethiopia; ^7^Department of Medical Laboratory Science, College of Medicine and Health Sciences, Arba Minch University, Arba Minch, Ethiopia

**Keywords:** knowledge, attitude, mental illness, Mattu, Ethiopia

## Abstract

**Background:**

The magnitude of mental health conditions in the general population was high in low-resource settings like Ethiopia. This was accompanied by little evidence on knowledge, attitudes, and related determinants in the general population. Therefore, the current survey is planned to assess the knowledge, attitude, and related factors of the community toward mental illness in Mattu, South West Ethiopia.

**Objectives:**

Our study aimed to assess the knowledge, attitude, and related factors of the community toward mental illness in Mattu, South West Ethiopia.

**Methods:**

A community-based cross-sectional survey was conducted in South West Ethiopia, Mattu town from 1 April−20 June, 2022 using a systematic random sampling, a multistage stratified technique from 649 households, and employed an interviewer-administered pre-tested semi-structured English version questionnaire. Epi-data Version 3.1 and SPSS-V-23.3 were employed for data entry and analysis respectively. A statistically significant association was declared at a *P*-value ≤ 0.05 at a 95% confidence interval.

**Results:**

In the current study, poor knowledge regarding, and unfavorable attitudes toward, mental illness among study respondents were 28% (182) 95% CI (24.3, 31.6) and 60.4% (392) 95% CI (56.5, 64.3), respectively. After controlling for potential confounders, being self-employed was independently associated with poor knowledge [AOR = 3.1, 95%CI (1.65, 4.28)]. Moreover, current use of substances [AOR = 1.64 95%CI (1.09, 5.98)] and not hearing information about mental illness from social media have been shown to be associated in the final model with an unfavorable attitude [AOR = 3.44 95%CI (1.98, 5.99)].

**Conclusion and recommendation:**

About one-third and more than one-half of the study participants showed poor knowledge and an unfavorable attitude, respectively. Compared to similar global and local findings, there was better community knowledge and a poor attitude toward mental illness in the area. Unfavorable attitudes toward mental illness were found to be exacerbated by participants not hearing about it on social media and by current substance use. Moreover, being self-employed was independently associated with poor knowledge of mental illness. Hence, all concerned stakeholders need to enhance mental health advocacy to improve public knowledge and attitude toward mental illness through media campaigns with a special focus on common substances. In addition, due attention should be given to self-employed groups of society to reduce the impacts of mental health conditions.

## Introduction

Mental health is a state of well-being in which an individual can realize one's own potential, cope with everyday stresses, work productively, and contribute to society (1). Mental health conditions are common in all regions of the world, affecting every community and age group across all income categories. Mental, neurological, and substance use disorders account for ~14% of the global disease burden (2) and one in every four people worldwide will be affected by a mental health condition at some point in their lives (3). More than 75% of patients with mental health conditions in many low-income countries do not have access to treatment (1).

The current prominence of mental illness is accompanied by a small number of mental health taskforces and related infrastructures such as hospital beds, with extreme variations when low-resource countries are considered. A WHO report shows that the number of mental health workers averages from below two workers per 100,000 people in low resource settings to more than 60 in high resource settings. Moreover, the average number of mental health beds again ranges below two in low resource settings for a similar number of people extending above twenty-five in high resource settings (4).

A research study conducted in rural areas of Ethiopia showed that the burden of mental illness encompasses about 11% of the total burden of disease, with depression and schizophrenia counted among the top 10 priorities of troublesome circumstances, out-ranking HIV/AIDS (4) which indicates that mental illness is still being neglected as a core health priority in Ethiopia.

The prevalence of mental health conditions in the general population was high in low resource settings like Ethiopia, as a report from different settings indicates, and this includes common mental illnesses (ranging from 21.6–27.9%) in 2018 (5), major depression (6.8%) (6), Khat use disorder (5%) (7), childhood psychiatric illness (12.5–22%) (8) and schizophrenia (0.5%) 2012–2016 (9). The crude suicide rates in Ethiopia are stated to have accelerated (7.9 to 8.4/100,000 population) over the last 10 years (2005–2015) (1). However, the actual suicide rate is likely to be substantially higher because of the stigma and taboos associated with suicide that lead to under-reporting.

Stigma and discrimination surrounding mental disorders represent not only factors that hamper treatment-seeking (10), but may also delay the healing process for people with mental disorders (11) and prevent people with mental disorders from achieving their social rights and full participation in the life of their community. It is known that knowledge and attitudes of the community toward mental illness have a significant role in the promotion and prevention of mental health conditions.

Earlier research reports show that poor knowledge and negative attitudes among general populations related to mental health are the foremost factors paving the way to stigma and discrimination among individuals living with mental disorders (12, 13). Moreover, studies showed sex, educational status, mental health training, economic status, and history of mental illness in the family were identified to predict negative attitudes among adults (14, 15).

The cumulative effects of negative views and stigmatization finally contribute to individuals with mental illness seeking professional support from healthcare institutions after they have attempted all available alternatives and the symptoms get worse (16, 17), which reduces the prognosis and outcome of the illness (18). Studies showed the stigmatizing nature of mental disorders was often related to poor knowledge (19, 20). In other words, the community's attitude toward mental illness was explained in terms of violence, erratic, and odd behavior; they were responsible for their condition, difficult to talk to, and incurable (21, 22).

A study conducted in different settings revealed a prevalence of poor knowledge about mental illness ranging from 3% in India to 69.1% in Lebanon (13–16) with positive attitude scores dominating in studies conducted in Lebanon (23) and South West Nigeria (24).

A study done in northeast Ethiopia showed the prevalence of poor knowledge and unfavorable attitudes toward mental illness were 55.3 and 45.1%, respectively (25). Additionally, another study reported inadequate knowledge of mental illness to be 44.8% (26). Research reveals that individuals living with mental illness experience stigma in their religious life, occupational areas, and social relationships, among other areas, which clearly indicates that negative perception and stigma impact more than the illness itself. Research reports have consistently shown that the knowledge and attitude of the public toward mental illness is crucial to effectively integrating mental health intervention (27).

An earlier study conducted in Ethiopia shows that the community's perspective supports the requirement for intervention for severe mental illnesses like schizophrenia and bipolar disorder. Since the causes of this mental illness are believed to be elucidated by traditional views rather than modern aspects, making traditional help (cultural or religious) the preferred modality of intervention compared to visiting healthcare institutions (20).

So, it's crucial to estimate the knowledge and attitude of the communities toward mental illness to accurately identify the negative perception, reduce the impacts of suffering, and design appropriate intervention related to mental illness in Ethiopia. Moreover, most of the earlier studies in Ethiopia focused on a certain segment of the population (18, 21) while the current study focused on the general population and a larger sample size compared to earlier studies in Ethiopia which is an asset for researchers.

Generally, despite the alarmingly rising burden of mental illness, there is still insufficient information that supports the evidence on the knowledge and attitude of the community in low-resource countries like Ethiopia, and particularly no previous studies in Mattu, Ethiopia. Therefore, the current survey assessed the knowledge and attitude of the community toward mental illness and its correlates among Mattu town residents, South West Ethiopia.

## Methods and materials

### Study design and setting

A community-based cross-sectional study was conducted among residents in Mattu town from 1 April−20 June, 2022. A study was done in the Ilu Aba Bor Zone, which is found in the South West Region of Ethiopia. Mattu town is the capital of Ilu Aba bor, located 600 km away in the south-western direction from Addis Ababa, the capital city of Ethiopia. Mattu has a total population of 14,056.

### Population

The source population of this study were all households in Mattu town, whereas the study population were all households residing in selected Kebeles (small administrative units) of Mattu town at the time of data collection. Individuals aged ≥18 and living in Mattu town for at least 6 months in selected Kebeles during the study period were included in the study. However, those individuals suffering from acute exacerbations of illness were excluded from this study.

### Sample size determination

The sample size for the study was determined by using single population proportion formula


$$\begin{array}{l} n=\frac{{\left[z\frac{\alpha }{2}\sqrt{p\left(1-p\right)}\right]}^{2}}{d^{2}} \end{array}$$

considering a 95% confidence level that falls within a 5% margin of error, 15% non-response rate, 1.5 design effect (because of clustering) (22) and knowledge, attitude, and associated factors toward mental illness among the community of Jimma town, Ethiopia (*P* = 44.8%). A total of 656 households were included in the study.

### Sampling procedures

**Figure 1:**
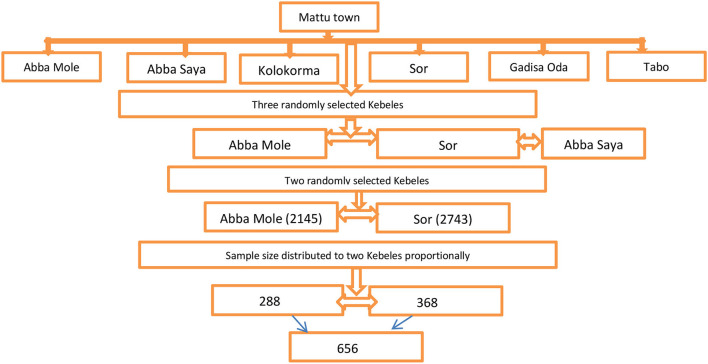
Schematic representation of sampling procedure of Mattu town Kebeles, Oromia Regional State, Ethiopia, 2022.

A multistage stratified sampling technique was applied to select study participants. In the first stage, considering a WHO standard for community-based studies, 30% of the total Kebeles were selected, and 2 Kebeles (Aba Saya and Sor) were selected randomly. The number of households available for the selected Kebeles was obtained from each of the Kebeles' offices. Accordingly, the calculated sample size was proportionally allocated to the two selected Kebeles. The households were selected through a systematic random sampling. The sampling interval (K) achieved was 5 (4,250/656 = 6.4 ≈ 6) so that households within the interval of 6 were surveyed by systematic random sampling. In the event that multiple households were found in a house, one household was selected by lottery method. Moreover, interviews were done by selecting one adult from a sampled household and using the lottery method when there was more than one adult in a single household (Figure 1).

### Data collection method, instrument, and quality control

Data was collected using an interviewer-administered pre-tested semi-structured English version questionnaire. Initially, the English-developed tool was translated back-to-back into the local languages Afaan, Oromo, and Amharic to ensure its suitability and consistency. A pre-test was performed on 20 (5%) of individuals residing in other Kebeles located in Ilu Aba Bor, which was not included for the current study. Data was collected through face-to-face interviews by three trained students graduating with a BSc in psychiatry from Mattu University and supervised by two senior lecturers. It included structured socio-demographic variables that were developed after an extensive review of various literature and related studies. The perceived level of social support was measured using the Oslo-3 social support scale. The instrument has shown good reliability (Cronbach α = 0.84) for this sampled population. It can be rated with scores ranging from (3–8), (9–11), and (12–14) as poor, moderate, and strong social support, respectively (28).

Community mental health knowledge and awareness of the participants was assessed using the Mental Health Knowledge Questionnaire (MHKQ). The instrument contains 20-items with two sections (1–16 items and 17–20) and self-administered in nature (Appendix 1). All items ranging from 1 to 16 are scored dichotomously as “yes and no” with various scores among items. Accordingly, items 1, 3, 5, 7, 8, 11, 12, 15, and 16 all correspond to 1 and 0 score for “yes” and “no” answers, respectively. Otherwise, for items 2, 4, 6, 9, 10, 13, and 14 a point score of 1 and 0 shows for “no” and “yes” answer, respectively. Items 17–20 were used to assess previous knowledge on the four mental health promotion days which include International Mental Health Day, International Day Against Drug Abuse and Illicit Drug Trafficking, International Suicide Prevention Day, and World Sleep Day. Generally the instruments have scores ranging from 0 to 20; higher scores show greater mental health knowledge (23). In the current study the Cronbach's coefficient for the MHKQ is 0.82.

The Community Attitude to Mental Illness Inventory (CAMI) was used to assess a respondent's attitude toward mental illness. The instrument contains four items with four sub-scales (benevolence, authoritarianism, social restrictiveness, and community mental health ideology). The items are rated as per the 5-likert scales: 1 = strongly disagree, 2 = disagree, 3 = neutral, 4 = agree, and 5 = strongly agree. It was validated (24) and has been used in several settings, including Ethiopia (29) and the Cronbach's coefficient of the instrument for this study is 0.72.The score of the attitude was dichotomized on the mean score of the respondents as favorable and unfavorable. Current substance use was assessed by a structured questionnaire and considered as the use of Khat, tobacco, alcohol, and cannabis in the last 3 months (25).

### Operational definitions

Poor knowledge – mean score of below 10 indicates poor knowledge.

Good knowledge – mean score of 10 and above shows good knowledge.

Favorable attitude – mean score of 120 and greater indicates favorable attitude.

Unfavorable attitude – mean score of < 120 indicates unfavorable attitude.

### Study variables

The dependent variables were knowledge and attitude toward mental illness. Explanatory variables were sex, age, marital status, religion, ethnicity, educational status, friends with mental illness, monthly income, occupation, perceived level of social support, information about mental illness from social media, family history of mental illness, friends with mental illness, and current substance use.

### Data processing and analyses

Data was entered into Epi-data v3.1 and analyzed by SPSS v23. Bivariate binary logistic regression was done to see the association of each independent variable with the outcome variable. Explanatory variables with a *p*-value ≤ 0.2 in the bivariate analysis were candidates for multivariate logistic regression. A multivariable logistic regression model was fitted and an adjusted odds ratio with 95% CI was calculated to identify the independent association with the outcome variable. *P*-value ≤ 0.05 was considered statistically significant.

### Ethical consideration

This study was conducted in accordance with the Declaration of Helsinki. The study protocol was reviewed and approved by the ethical review board of the College of Health Science, Mattu University (Approval ID: ARCSV/280/14). Informed consent was obtained from each study participant. Names of participants and other personal identifiers were not included in the data collection tool, and participants' consent included the publication of anonymous responses. Confidentiality was kept to the utmost and participation was totally voluntary, with no incentive or other benefit.

## Result and discussion

A total of 656 respondents were sampled in the study, and 649 successfully responded to the questionnaires, making an overall response rate of 98.9%. Considering gender, males have a slightly higher preponderance 355 (54.7%) and most of the participants' age range lies between 26 and 35 years. More than half 354 (54.5%) were married and nearly 623 (73.8%) were self-employed (Table 1).

**Table 1:** Socio-demographic, clinical and psychosocial characteristics of Mattu town residents, South West Ethiopia, 2022 (*n* = 649).

**Variables**	**Category**	**Frequency (N)**	**Percent (%)**
Sex	Male	355	54.7
	Female	294	45.3
Age in years	18–25	149	23.0
	26–35	252	38.8
	36–45	108	16.6
	≥46	140	21.6
Marital status	Single	231	35.6
	Widowed/divorced	64	9.9
	Married	354	54.5
Religion	Orthodox	182	28.0
	Muslim	203	31.3
	Protestant	232	35.7
	Catholic and others	32	4.9
Ethnicity	Oromo	510	78.6
	Amhara	54	8.3
	Tigre	19	2.9
	Other*	66	10.2
Educational status	Unable to read and write	100	15.4
	Able to read and write	180	27.7
	Primary (1–8)	68	10.5
	Secondary (9–12)	126	19.4
	College and above	175	27.0
Occupation status	Self- employed	491	75.7
	Government employed	158	24.3
Average monthly income of the household	< 2,300	455	70.1
	≥2,300	194	29.9
Family history of mental illness	Yes	11	–
	No	638	98.3
Have you heard about mental illness from social media	Yes	198	30.5
	No	451	69.5
Do you have friends suffer mental illness	Yes	19	2.9
	No	630	97.1
Current use of substance (Khat, alcohol, cigarette, and cannabis)	Yes	454	70.0
	No	195	30.0
Social support	Poor	435	67.0
	Moderate	186	28.7
	Strong	28	4.3
Mental health knowledge	Poor	182	28.0
	Good	467	72.0
Attitude toward mental illness	Favorable	392	60.4
	Unfavorable	257	39.6

*Silte, Gurage, Wolaita, Dawuro, Agnuak, Nuer, Mazang.

The majority of the study respondents earned < 2,300 ETB (70%) (Ethiopian birr) average income on a monthly basis. Only about one-third of them had heard about mental illness from social media, 198 (30.5%), and the majority of the respondents currently used khat, cigarettes, and alcohol in the past 3 months.

### Prevalence of knowledge toward mental illness among study respondents

**Figure 2:**
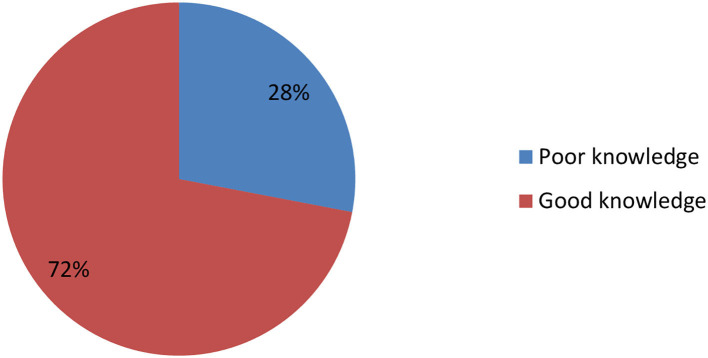
Knowledge toward mental illness among study respondents among Mattu town residents, South West Ethiopia, 2022 (*N* = 649).

In the current study, respondents' knowledge of mental illness was assessed using the Mental Health Knowledge Questionnaire (MHKQ) instrument, with a mean score of < 10. Accordingly, about one-fourth of the study respondents, 182 (28%) (95% CI (24.3, 31.6), reported poor knowledge of mental illness (Figure 2).

#### Factors associated with knowledge toward mental illness among study respondents

In the binary logistic regression, knowledge of mental illness was found to be higher among those with a higher educational status, age of the respondents, hearing about mental illness from social media, having friends with mental illness, marital status, and occupational status. However, after controlling for potential confounders in the multivariable logistic regression, self-employment [AOR = 3.1, 95%CI (1.65, 4.28)] was shown to have an association in the final model with poor knowledge of mental illness (Table 2).

**Table 2:** Bivariate and multivariate logistic regression analysis of factors associated with poor knowledge toward mental illness among Mattu town residents, South West Ethiopia, 2022 (*N* = 649).

**Variable**	**Category**	**Poor knowledge**		**COR,95% (CI)**	**AOR,95% (CI)**
		**Yes *N* (%)**	**No *N* (%)**		
Educational status	Unable to read and write	35 (35.0)	65 (65.0)	0.43 (0.25,0.76)	0.305 (0.15,0.62)
	Can read and write	54 (30.0)	126 (70.0)	0.54 (0.33,0.89)	0.36 (0.21, 0.62)
	1–8	15 (22.1)	53 (77.9)	0.82 (0.41,1.63)	0.79 (0.34,1.89)
	9–12	45 (35.7)	81 (64.3)	0.42 (0.25,0.71)	0.56 (0.29,1.09)
	College and above	33 (18.9)	142 (81.1)	1	1
Age of the respondents	18–25	50 (33.6)	99 (66.4)	0.85 (0.52, 1.39)	0.19 (0.07, 1.67)
	26–35	66 (26.2)	186 (73.8)	1.21 (0.76,1.91)	0.99 (0.55, 1.82)
	36–45	24 (22.2)	84 (77.8)	1.50 (0.84,2.68)	1.18 (0.62,2.27)
	≥46	42 (30.0)	98 (70.0)	1	1
Heard about mental illness from social media	Yes	63 (31.8)	135 (68.2)	1	
	No	119 (26.4)	332 (73.6)	1.30 (0.90,1.88)	1.01 (0.61, 1.67)
Having friends with mental illness	Yes	63 (31.8)	135 (68.2)	1	1
	No	119 (26.4)	332 (73.6)	1.96 (0.90, 1.87)	1.90 (0.72, 5.05)
Occupational status	Self employed	121 (24.6)	370 (75.4)	0.72 (0.59,0.87)	3.10 (1.67, 5.77)**
	Government employed	61 (38.6)	97 (61.4)	1	1
Marital status	Single	56 (24.2)	175 (75.8)	1	1
	Widowed/divorced	19 (29.7)	45 (70.3)	0.76 (0.41,1.40)	0.08 (0.21,0.28)
	Married	182 (28.0)	467 (72.0)	0.74 (0.51,1.08)	0.12 (0.04,0.30)

**p < 0.001, reference category =1. The bold values indicate the variable associated in the final regression model.

### Attitude toward mental illness among study respondents

**Figure 3:**
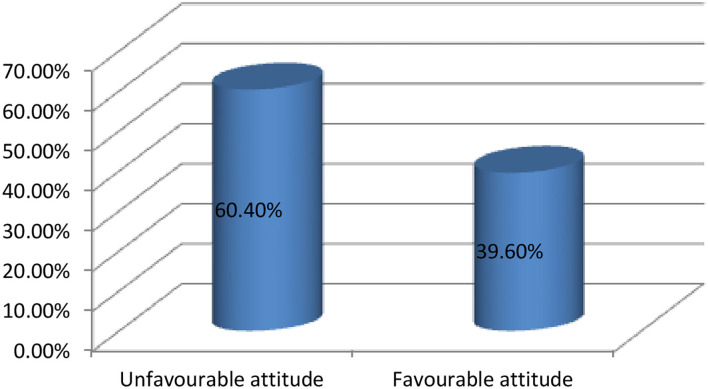
Attitude of the study respondents toward mental illness among Mattu town residents, South West Ethiopia, 2022.

The attitude of the study respondents toward mental illness was measured using the Community Attitude to Mental Illness Inventory (CAMI) instrument using a mean score of 120. Accordingly, more than half of the study respondents, 60.4% (392), 95% CI (56.5, 64.3), showed an unfavorable attitude toward mental illness (mean score of below 120) (Figure 3).

#### Factors associated with attitude toward mental illness among study respondents

In the bi-variable logistic regression, unfavorable attitude toward mental illness was found to be associated with gender, educational status, age of the respondents, marital status, family history of mental illness, hearing about mental illness from social media, having friends with mental illness, current use of substances, monthly income, and occupational status of the study participants.

However, after controlling for potential confounders in the multivariable logistic regression, only a couple of covariates, including not hearing about mental illness from social media [AOR = 3.44 95%CI (1.98, 5.99)], and current use of substances [AOR = 1.64 95%CI (1.09, 5.98)] were associated in the final model with an unfavorable attitude toward mental illness (Table 3).

**Table 3:** Bivariate and multivariate logistic regression analysis of factors associated with unfavorable attitude toward mental illness among Mattu town residents, South West Ethiopia, 2022 (*N* = 649).

**Variable**	**Category**	**Unfavorable attitude**		**COR,95% (CI)**	**AOR,95% (CI)**
		**No *N* (%)**	**Yes *N* (%)**		
Gender	Male	198 (55.8)	157 (44.3)	1	1
	Female	194 (66.0)	100 (34.0)	0.65 (0.47,0.89)	0.86 (0.59,1.24)
Educational status	Unable to read and write	82 (82.0)	18 (18.0)	0.19 (0.10, 0.35)	0.48 (0.23, 0.98)
	Can read and write	112 (62.2)	68 (37.8)	0.54 (0.35,0.82)	0.68 (0.41,1.12)
	1–8	47 (69.1)	21 (30.9)	0.39 (0.22,0.71)	0.30 (0.14,0.66)
	9–12	69 (54.8)	57 (45.2)	0.73 (0.46,1.15)	0.64 (0.36,1.15)
	College and above	82 (46.9)	93 (53.1)	1	1
Age of the respondents	18–25	99 (66.4)	50 (33.6)	0.83 (0.51, 1.34)	0.19 (0.08,0.46)
	26–35	138 (54.8)	114 (45.2)	1.36 (0.89,2.07)	0.53 (0.29,0.94)
	36–45	68 (63.0)	40 (37.0)	0.97 (0.58,1.62)	0.79 (0.43,1.45)
	≥46	87 (62.1)	53 (37.9)	1	1
Marital status	Single	129 (55.8)	102 (44.3)	1	1
	Widowed/divorced	46 (71.9)	18 (28.1)	0.49 (0.27,0.90)	0.69 (0.27,1.79)
	Married	217 (61.3)	137 (38.7)	0.79 (0.57,1.12)	0.59 (0.33,1.08)
Family Hx of mental illness	Yes	10 (90.9)	1 (9.1)	1	1
	No	382 (59.9)	256 (40.1)	6.70 (0.85,52.7)	3.06 (0.37,25.54)
Heard about mental illness from social media	Yes	164 (82.8)	34 (17.2)	1	1
	No	228 (50.6)	223 (49.4)	4.72 (3.12,7.13)	3.44 (1.98,5.99)**
Current use of substance	Yes	310 (68.3)	144 (31.7)	1	1
	No	82 (42.1)	113 (57.9)	2.97 (2.09,4.19)	1.64 (1.09,5.98)*
Monthly income	< 2,300	291 (64.0)	164 (36.0)	0.61 (0.44,0.86)	0.81 (0.55,1.19)
	≥2,300	101 (52.1)	93 (47.9)	1	1
Occupational status	Self employed	321 (65.4)	170 (34.6)	1.52 (1.27,1.83)	0.53 (0.29, 0.93)
	Government employed	71 (44.9)	87 (55.1)	1	1

*p < 0.05, **p < 0.001, reference category = 1, Hx, history. The bold values indicate the variable associated in the final regression model.

## Discussion

The stigma surrounding mental illness, which is a significant impediment to treatment seeking and a contributor in the poor prognosis for those who suffer from it, could conceivably be reduced by the communities' increased awareness of the condition (30–32). Likewise, the community's favorable perception of mental illness has a significant impact on the lives of survivors (26). For instance, the survivors' treatment-seeking behaviors and the perception of the treatment outcomes vastly depend on the perceptions and attitudes of their families or significant others, as well as their communities at large (30, 33). Baseline data regarding the communities' knowledge and attitude toward mental illness is fundamental to endorsing and strengthening the management and prevention strategies. Therefore, the present study aimed to assess the knowledge and attitude of the community toward mental illness and predictors in Mattu town, South West Ethiopia.

In our study, more than one-quarter (28%) of the participants showed poor knowledge of mental illness. The current finding is lower than the previous studies showing poor knowledge conducted in Jimma (44.8%), Dessie Town (55.3%), South West Nigeria (51.2%), Southwest Cameroon (32.1%), Lebanese (67%), and the Fiji Islands (35%) respectively (23–26, 29, 32). Variations in the sociodemographics and traditional values of the communities may be responsible for this disparity. The present finding is lower than a study from Gadag, India, where 3% of participants had poor knowledge (34). This difference might be due to the small sample size utilized in the previous study. In addition, sociodemographic differences between the populations could be a reason for this significance. The two discrepancies could also be due to ethnic differences between the study communities as evidenced by Bener and Ghuloum's study (35).

In this study, being self-employed was shown to have a significant association with the participants' poor knowledge of mental illness. Accordingly, the odds of poor knowledge of mental illness were three-fold higher among self-employed participants than among government employees. The general public was not cognizant of this significant association. However, a study conducted among health extension professionals in Addis Ababa found a significant association between the absence of job aid and poor knowledge of mental illness (36, 37). The possible reason for this association might be explained by poor exposure to information about mental illness in the absence of some government sponsored training. Moreover, the discrepancies in the study populations may be responsible for this disparity.

The current study found that 60.4% of participants had an unfavorable attitude toward mental illness. This finding is significantly higher than a study conducted in Gadag, India, in which none of the study participants showed an unfavorable attitude (14). The present finding is higher than a study conducted in Dessie town (45.1%), the Southwest Region of Cameroon (37.7%), South West Nigeria (10%), and Saudi Arabia (18%) (24, 25, 29, 38–41). These dissimilarities might be linked to differences in socio-cultural and traditional attributes of the study populations. However, the present finding is lower than a study conducted in Lebanon (67.8%) (23). The possible reason for this difference could be the religious and cultural variations in the previous and latter study populations. The significant negative attitude reported in this study has not been linked to the categories with higher levels of knowledge. Previous research has found that a lack of knowledge contributes to a negative attitude, which leads to stigma and discrimination of mental health conditions. The current study also confirmed the absence of an association between good knowledge and a negative attitude.

In the contemporary world, the media's influence on mental illness is not restricted to delighting in the negative aspects but rather creates a positive attitude toward mental health awareness. In this study, hearing information about mental illness from social media was shown to have a significant association with the attitude toward mental illness. The likelihood of having an unfavorable attitude was more than three times higher among participants who had not heard about mental illness from social media than among those who had. The community's cultural impact, morals, and beliefs in the conventional causes of mental illness may be the cause, and might also be accountable for the negative attitude toward mental illness (27, 42).

Additionally, individuals' attitudes on mental illness in the current study were highly correlated with their substance use behavior. Participants who were not using substances were 1.6-times more likely to have an unfavorable attitude than participants who were using substances. This was a novel association that had not been identified in earlier studies. It might be related to the censuring of mentally ill people by the non-user participants who perceive substance-using behavior as a cause of mental illness (43).

Nearly all of the participants in this study who had a family history of mental illness had a positive attitude regarding mental illness. This was consistent with a previous study (Goffman, 1963) that connected a negative attitude with a relative's lack of a history of mental illness. This could be explained by the idea that interacting with people who were mentally ill gave them new perspectives on having a positive attitude.

The community-based study nature and the utilization of a large sample size could be the strength of the contemporary study, whereas the descriptive and cross-sectional nature of this study could be taken as its shortfall because it could not assert cause-effect relationships as well as dig out the communities' ingrained perceptions and attitudes toward mental illness. Hence, we recommend future interested researchers use more comprehensive study designs.

## Conclusion

About one-third and more than one-half of the study participants showed poor knowledge and unfavorable attitudes, respectively. Compared to similar global and local findings, there was better community knowledge and attitude toward mental illness in the area. Not hearing about mental illness from social media and current use of substances were found to be enhancing factors for unfavorable attitudes toward mental illness. Moreover, being self-employed was independently associated with poor knowledge of mental illness. Hence, the government and concerned stakeholders need to advocate on the dominant social media about mental illness to enhance public knowledge and attitude toward mental illness through media campaigns. In addition, they need to also better target self-employed segments of society to reduce the impact of mental health conditions and scale-up public mental health awareness at large.

## Data availability statement

The datasets presented in this study can be found in online repositories. The names of the repository/repositories and accession number(s) can be found in the article/Supplementary material.

## Ethics statement

The studies involving human participants were reviewed and approved by College of Health Science, Mattu University (Approval ID: ARCSV/280/14). The patients/participants provided their written informed consent to participate in this study.

## Author contributions

MJ, MM, and AM wrote and designed the protocol, led the data collection process, analyzed the data, and reviewed and edited the manuscript. GD, WG, and DN revises and approves the protocol, takes part in data analysis, reviews, and edits the manuscript. AM, MH, and KJ contributed to data analysis, drafting the manuscript, critically reviewing, and approving the manuscript for publication. All authors read and approved the final manuscript.

## Conflict of interest

The authors declare that the research was conducted in the absence of any commercial or financial relationships that could be construed as a potential conflict of interest.

## Publisher's note

All claims expressed in this article are solely those of the authors and do not necessarily represent those of their affiliated organizations, or those of the publisher, the editors and the reviewers. Any product that may be evaluated in this article, or claim that may be made by its manufacturer, is not guaranteed or endorsed by the publisher.
