# Supplementary figures and images for: Knowledge, attitude, and its correlates of the community toward mental illness in Mattu, South West Ethiopia
Source: Front Psychiatry. 2022 Nov 8;13:1018440. doi: 10.3389/fpsyt.2022.1018440 (PMC9679014; doi:10.3389/fpsyt.2022.1018440)

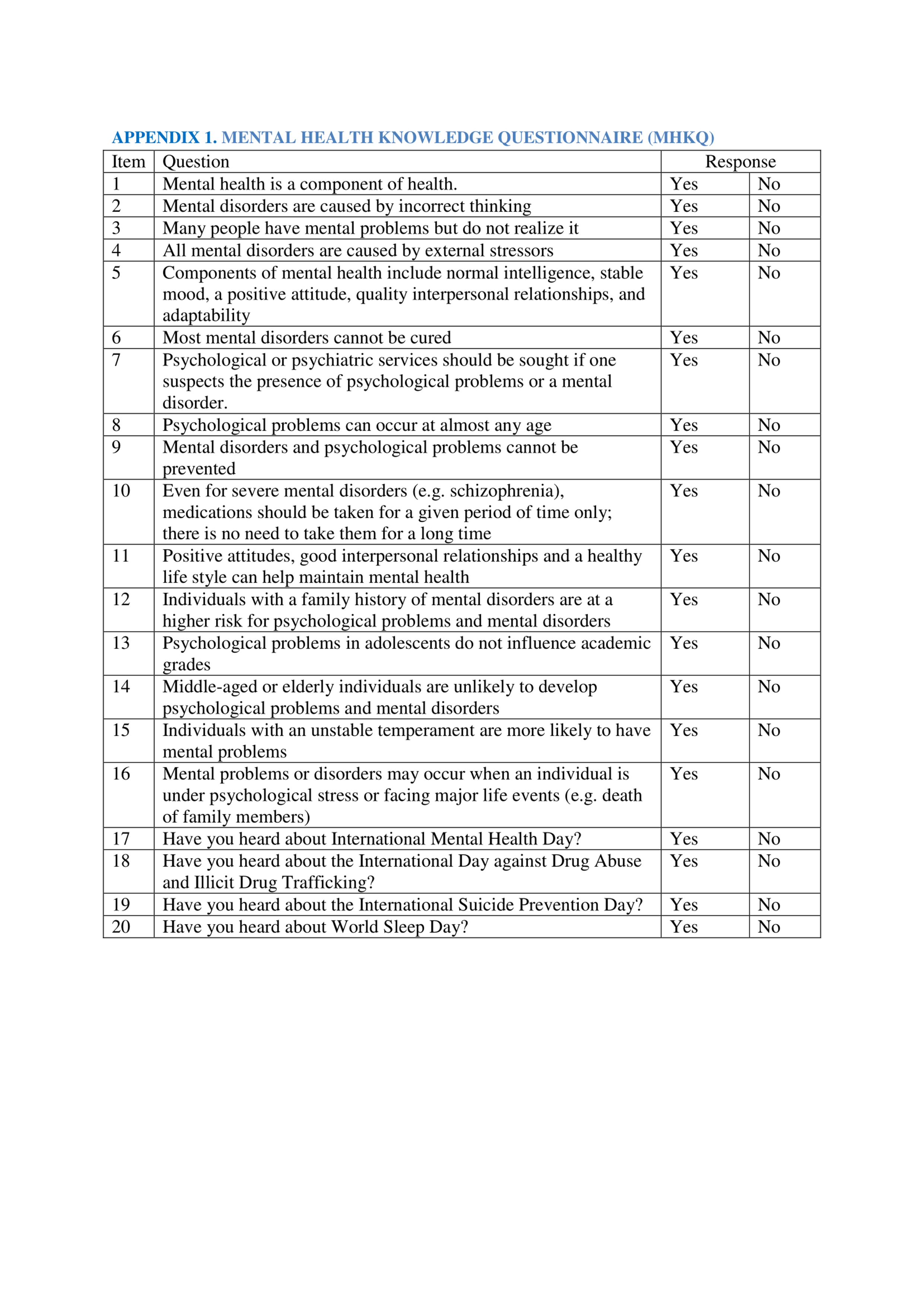

Supplement: Supplementary file 1 [file Image_1.TIFF]
